# Edoxaban versus Apixaban Outcomes Differences in 8,444 Patients with Atrial Fibrillation from Italy: A Real-World Use Comparison

**DOI:** 10.1055/a-2822-9746

**Published:** 2026-03-23

**Authors:** Angel Valladares, Joseph Imperato, Sidharth Gupta, Rosa Wang, Shantanu Jawla, Matthew Clasen, Rüdiger Smolnik, Cathy Chen, Martin Unverdorben, Xin Ye, Giuseppe Patti, Bernd Brüggenjürgen

**Affiliations:** 1 IQVIA, New York, New York, United States; 2 IQVIA, Bengaluru, Karnataka, India; 3 Daiichi Sankyo Inc., Basking Ridge, New Jersey, United States; 4 Daiichi Sankyo Europe GmbH, Munich, Germany; 5 Department of Translational Medicine, Università del Piemonte Orientale, Novara, Italy; 6 Orthopedic Department, Institute for Health Services Research and Technical Orthopedics, Hannover Medical School at DIAKOVERE Annastift Hospital, Hannover, Germany

**Keywords:** atrial fibrillation, anticoagulant, thrombosis, real-world studies

## Abstract

**Background:**

Based on efficacy and safety data from randomized controlled trials (RCTs) and real-world studies, direct oral anticoagulants are recommended for thromboembolism prevention in patients with atrial fibrillation (AF). However, no RCTs have compared edoxaban with apixaban.

**Objectives:**

This study aimed to retrospectively compare clinical outcomes for patients with AF in Italy (overall and by age group) who received edoxaban versus apixaban.

**Methods:**

Adult patients with AF who newly initiated an edoxaban or apixaban prescription between January 2016 and December 2021 were identified from the Italian IQVIA ^®^ Longitudinal Patient Database. Patient characteristics were summarized. Propensity score matching was used to balance baseline characteristics between the edoxaban and apixaban groups. Clinical outcomes of effectiveness (ischemic stroke [IS] or systemic embolism [SE]) and safety (any major bleeding [MB]) were compared. Incidence rates per 100 person-years and hazard ratios (HRs) with 95% CIs were computed.

**Results:**

Among 8,444 identified patients, 37.8% ( *n* = 3,188) were prescribed edoxaban and 62.2% ( *n* = 5,256) were prescribed apixaban. After matching, patient characteristics were similar between cohorts. The post-matching risk (HR, 95% CI) of IS/SE was significantly lower for edoxaban versus apixaban in the overall population (0.78, 0.61–0.99; *p* = 0.04) and in patients aged ≥80 years (0.61, 0.44–0.86; *p* < 0.01), with a similar risk for MB for edoxaban versus apixaban. No significant differences were observed between edoxaban and apixaban among patients aged <80 years (all *p* > 0.05).

**Conclusion:**

IS/SE risk was significantly lower for edoxaban versus apixaban, without an increased MB risk among patients with AF overall and those aged ≥80 years.

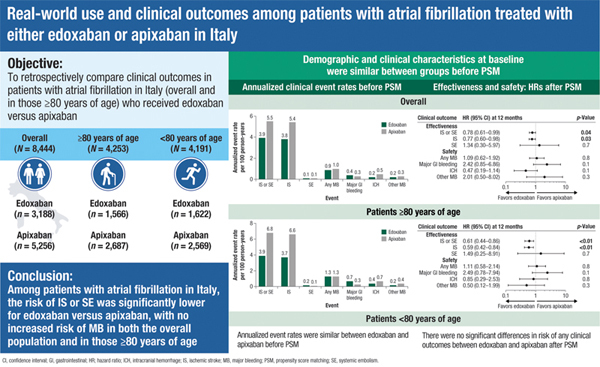

## Introduction

Atrial fibrillation (AF) affects approximately 59 million people worldwide, significantly increasing the risk of ischemic stroke (IS), heart failure, and mortality. 1 2 3 The global prevalence of AF has risen over the past two decades (currently estimated at 2–4%) and is expected to continue increasing due to an aging population and improved diagnostic capabilities. 4 5 In Italy, specifically, 13.7% of individuals aged 80 to 84 years and 16.1% of those aged ≥85 years had AF in 2016. 6

Direct oral anticoagulants (DOACs) have become the standard for thromboembolic prevention in AF, replacing vitamin K antagonists (VKAs) in clinical practice. 5 7 8 9 10 11 Apixaban, dabigatran, and rivaroxaban were the first DOACs approved by the Italian Drug Agency (AIFA) in 2013, followed by edoxaban in 2016. 12 13 14 15 While apixaban is the most prescribed DOAC in Italy, 16 edoxaban is the least prescribed; however, edoxaban prescription has been steadily increasing since 2017, reflecting a growing preference for this anticoagulant. 17 Despite its availability for nearly a decade, real-world data directly comparing edoxaban and apixaban in Italian patients with AF are lacking.

The efficacy and safety of both edoxaban and apixaban compared with warfarin for thromboembolic stroke prevention in AF were established in their respective pivotal randomized controlled trials (RCTs; ENGAGE AF-TIMI 48 for edoxaban and ARISTOTLE for apixaban). 9 11 There are currently no published or planned head-to-head RCTs comparing edoxaban and apixaban, leaving real-world evidence as the primary source of data to inform clinical decision-making. 8 11 Such comparative insights would be valuable not only within an overall patient with AF population, but also in patients ≥80 years of age, who face heightened risks of both any stroke 18 and bleeding. 19 20 Older patients also exhibit age-related pharmacokinetic and pharmacodynamic changes that can influence anticoagulant effects. 21 To address this gap, the present retrospective cohort study compares the real-world use and clinical outcomes of edoxaban and apixaban among patients with AF in Italy and also within subpopulations of individuals <80 and ≥80 years of age. By analyzing real-world evidence, this study aims to provide health care professionals with comparative data to support informed anticoagulant selection.

## Methods

### Data Source

Retrospective patient data spanning the study period (January 1, 2016, to December 31, 2021) were obtained from the Italian IQVIA ^®^ Longitudinal Patient Database. This database comprises anonymized patient data from general practitioners (GPs; e.g., data on patients' consultations and treatments).

### Study Sample

Adult patients with AF who were newly prescribed edoxaban (30 and 60 mg) or apixaban (2.5 and 5 mg) during the study period were identified. The index date for each patient was the index prescription of a new edoxaban or apixaban prescription. Patients were eligible if they had 12 months of data prior to the index prescription. Patients were excluded if they had a prescription for any VKA or DOAC within 12 months prior to the index prescription. Patients were excluded if they had a diagnosis of moderate to severe mitral stenosis or a mechanical valve replacement within the 12 months preceding their index date. 22 Patients were also excluded if they had a deep vein thrombosis or pulmonary embolism diagnosis within the 12 months before their index date. The remaining patients were considered representative of an overall population with AF in Italy.

### Follow-Up Period

The follow-up period for which clinical outcomes were measured was the time from the patient's index date until (1) 12 months after the index prescription, (2) the first effectiveness or safety outcome event, (3) change of treatment, or (4) death, whichever occurred first.

### Baseline Characteristics

Patient demographic and clinical characteristics were recorded at baseline. Demographic variables included age and sex. Clinical characteristics included the CHADS _2_ (Congestive heart failure, Hypertension, Age ≥75, Diabetes, Stroke [doubled]) score, CHA _2_ DS _2_ -VASc (Congestive heart failure, Hypertension, Age ≥75 [doubled], Diabetes, Stroke [doubled], Vascular disease, Age 65–74 years, and Sex category [female]) score, and Charlson Comorbidity Index (CCI). The CHADS _2_ and CHA _2_ DS _2_ -VASc scores are estimates for predicting the risk of IS in patients with AF. 23 Prior use of at least one of the following prescription medications was considered concomitant medication: Antiplatelet medication, nonsteroidal anti-inflammatory drugs, H _2_ -receptor antagonists, proton pump inhibitors, angiotensin-converting enzyme inhibitor/angiotensin receptor blocker, amiodarone, dronedarone, β-blocker, statins, etc.

### Outcomes

Effectiveness outcomes included combined IS or systemic embolism (SE), as well as individual IS and SE event rates per 100 person-years. Safety outcomes included major bleeding (MB), major gastrointestinal (GI) bleeding, intracranial hemorrhage (ICH), or other MB event rates per 100 person-years, as defined by Cunningham et al. 24 All event data were obtained from GP medical records.

### Statistical Analysis

#### Baseline Demographics and Clinical Characteristics Analyses

Categorical variables were summarized using frequencies and percentages, while continuous variables were summarized using means and standard deviations (SDs). Pearson's chi-square tests were employed for categorical variables, and *t* -tests were used for continuous variables, assuming normal distribution, to determine statistical significance between the edoxaban and apixaban cohorts in the overall population and among patients aged ≥80 years. For all statistical comparisons, a two-sided *p* -value of <0.05 was considered significant.

#### Propensity Score Matched Analyses

Propensity score matching was performed to adjust for confounding factors by separately matching (with a calliper width <20% of the SD) the edoxaban cohort to the apixaban cohort 1:1 based on demographic and baseline clinical characteristics. Matching was assessed by covariate (sex, age at index prescription, CHA _2_ DS _2_ -VASc score, CCI score, bleeding history, and concomitant medications) distribution before and after, standardized differences (Cohen's *d* ) and variance ratios, and graphical inspection of distributions.

Annualized clinical event rates were calculated before and after matching, using the formula below. Inferential statistics were not conducted on the annual clinical event rates.



After matching, multivariable Cox regression models were used to evaluate the risk of IS or SE and any MB. Hazard ratios (HRs), 95% confidence intervals (CIs), and *p* -values (calculated using the Wald test) for the reported outcomes were computed at 12 months between the edoxaban cohort and the apixaban cohort and for the subgroups of patients aged <80 or ≥80 years. All analyses were performed using Python ^®^ version 3.0.

## Results

### Baseline Characteristics

**Fig. 1:**
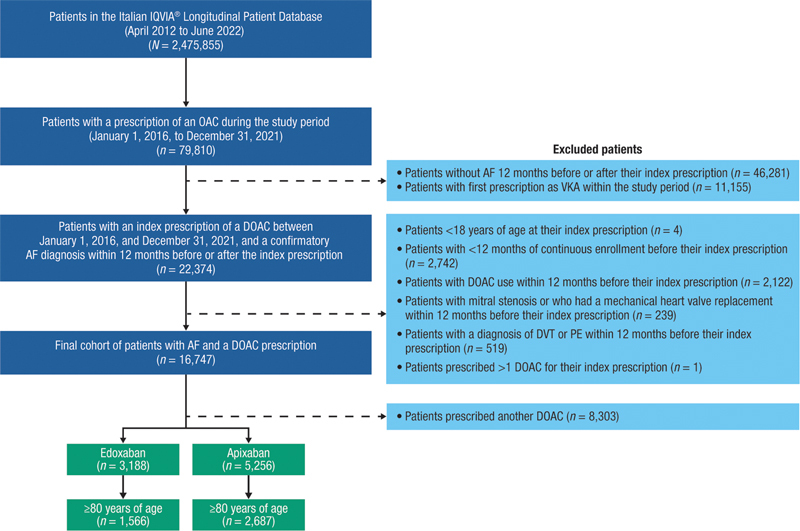
Flow chart of patient selection. AF, atrial fibrillation; DOAC, direct oral anticoagulant; DVT, deep vein thrombosis; OAC, oral anticoagulant; PE, pulmonary embolism; VKA, vitamin K antagonist.

Overall, 8,444 patients were eligible, with 37.8% ( *n* = 3,188) prescribed edoxaban and 62.2% ( *n* = 5,256) prescribed apixaban before matching ( Fig. 1 , Table 1 , and Supplementary Table S1 ). Approximately half of the patients were female in both the edoxaban and apixaban cohorts (53.6% and 52.2%, respectively). Among patients aged ≥80 years, 36.8% ( *n* = 1,566/4,253) were prescribed edoxaban and 63.2% ( *n* = 2,687/4,253) were prescribed apixaban. In this subpopulation, 60.1% in the edoxaban cohort, compared with 58.8% in the apixaban cohort, were female. The majority of patients in the edoxaban cohort (99.9%; *n* = 3,187/3,188) were retained after matching to the apixaban cohort, whereas 60.6% ( *n* = 3,187/5,256) of the apixaban cohort were retained after matching to the edoxaban cohort.

**Table 1:** Baseline demographics and clinical characteristics before matching among patients overall and those aged ≥80 years a b

	Overall ( *N* = 8,444)			aged ≥80 years ( *N* = 4,253)		
	Edoxaban ( *n* = 3,188)	Apixaban ( *n* = 5,256)	*p-* Value c	Edoxaban ( *n* = 1,566)	Apixaban ( *n* = 2,687)	*p* -Value c
**Demographics**						
Age, years						
Mean (SD)	78.1 (9.8)	78.5 (9.7)	0.1	85.9 (4.3)	85.9 (4.3)	0.6
Median (Q1, Q3)	79.0 (72.0, 85.0)	80.0 (73.0, 85.0)	–	85.0 (82.0, 89.0)	85.0 (82.0, 89.0)	
Age category (years)						
≤ 64	275 (8.6)	445 (8.5)	**0.02**	0	0	–
65–74	769 (24.1)	1,137 (21.6)		0	0	
≥ 75	2,144 (67.3)	3,674 (69.9)		1,566 (100.0)	2,687 (100.0)	
Sex						
Female	1,710 (53.6)	2,743 (52.2)	0.2	941 (60.1)	1,580 (58.8)	0.4
**Clinical characteristics**						
CHADS _2_ score, mean (SD)	2.0 (0.9)	2.0 (0.9)	**<0.0001**	2.3 (0.7)	2.4 (0.8)	**0.04**
CHA _2_ DS _2_ -VASc score, mean (SD)	3.5 (1.2)	3.6 (1.2)	**0.0007**	4.0 (0.9)	4.1 (1.0)	**0.05**
CCI score						
Mean (SD)	0.7 (1.1)	0.9 (1.2)	**<0.0001**	0.8 (1.1)	1.0 (1.3)	**<0.0001**
CCI score category			**<0.0001**			**0.0006**
0	1,775 (55.7)	2,666 (50.7)	–	817 (52.2)	1,276 (47.5)	–
1	848 (26.6)	1,484 (28.2)		439 (28.0)	783 (29.1)	
2	325 (10.2)	630 (12.0)		191 (12.2)	329 (12.2)	
>2	240 (7.5)	476 (9.1)		119 (7.6)	299 (11.1)	
Hypertension	2,928 (91.8)	4,830 (91.9)	0.9	1,464 (93.5)	2,477 (92.2)	**0.1**
Diabetes mellitus	623 (19.5)	1,097 (20.9)	0.1	314 (20.1)	543 (20.2)	0.9
Vascular disease	235 (7.4)	521 (9.9)	**<0.0001**	123 (7.9)	273 (10.2)	**0.01**
Coronary artery disease	149 (4.7)	375 (7.1)	**<0.0001**	70 (4.5)	190 (7.1)	**0.0006**
Peripheral artery disease	106 (3.3)	179 (3.4)	0.8	62 (4.0)	100 (3.7)	0.7
Stroke/transient ischemic attack	146 (4.6)	324 (6.2)	**0.002**	84 (5.4)	206 (7.7)	**0.004**
Congestive heart failure	243 (7.6)	442 (8.4)	0.2	144 (9.2)	285 (10.6)	0.1
Cerebral vascular disease	291 (9.1)	594 (11.3)	**0.002**	171 (10.9)	358 (13.3)	**0.02**
Chronic pulmonary disease	263 (8.3)	535 (10.2)	**0.003**	129 (8.2)	295 (11.0)	**0.004**
Cancer	180 (5.7)	314 (6.0)	0.5	83 (5.3)	183 (6.8)	**0.05**
Bleeding history or predisposition	19 (0.6)	48 (0.9)	0.1	11 (0.7)	26 (1.0)	0.4
Medications						
Antiplatelet	323 (10.1)	691 (13.2)	**<0.0001**	187 (11.9)	390 (14.5)	**0.02**
NSAIDs	275 (8.6)	437 (8.3)	0.6	127 (8.1)	212 (7.9)	0.8
Proton pump inhibitors	1,446 (45.4)	2,507 (47.7)	**0.04**	743 (47.5)	1,351 (50.3)	0.07
ACEI-ARB	1,361 (42.7)	2,428 (46.2)	**0.002**	682 (43.6)	1,228 (45.7)	0.2
Amiodarone	294 (9.2)	542 (10.3)	0.1	126 (8.1)	269 (10.0)	**0.03**
β-blocker	1,828 (57.3)	3,107 (59.1)	0.1	860 (54.9)	1,538 (57.2)	0.1
Statins	1,125 (35.3)	1,920 (36.5)	0.2	522 (33.3)	943 (35.1)	0.2

Abbreviations: ACEI-ARB, angiotensin-converting enzyme inhibitor-angiotensin receptor blocker; CCI, Charlson Comorbidity Index; CHADS _2_ , Congestive heart failure, Hypertension, Age ≥75, Diabetes, Stroke (doubled); CHA _2_ DS _2_ -VASc, Congestive heart failure, Hypertension, Age ≥75 (doubled), Diabetes, Stroke (doubled), Vascular disease, Age 65 to 74, and Sex category (female); NSAID, nonsteroidal anti-inflammatory drug; Q1, first quartile; Q3, third quartile; SD, standard deviation.

Bold values indicate *p* <0.05.

a Data are shown as *n* (percentage) unless otherwise noted.

b Baseline clinical characteristics <5% are not shown.

c *p* -Values indicate comparison to the edoxaban cohort.

Among patients aged ≥80 years, all patients in the edoxaban (100.0%; *n* = 1,566/1,566) cohort were retained after matching to the apixaban cohort, whereas 58.3% ( *n* = 1,566/2,687) of the apixaban cohort was retained after matching to the edoxaban cohort. Among patients aged <80 years, all but one patient in the edoxaban (99.9%; *n* = 1,621/1,622) cohort were retained after matching to the apixaban cohort; 63.1% ( *n* = 1,621/2,569) of patients in the apixaban cohort were retained after matching to the edoxaban cohort. For the overall population and both age-based subgroups, covariates were similar between the matched cohorts, with all standardized mean differences ≤0.1. Baseline demographic and clinical characteristics after matching are provided in Supplementary Table S2 .

### Pre-Matching Clinical Outcomes

The pre-matching annualized event rates of clinical outcomes for the overall population and the subgroups of patients aged ≥80 and <80 years are reported in Supplementary Tables S3 and S4 .

### Post-Matching Clinical Outcomes

#### Overall Population

For the overall population, edoxaban versus apixaban (3.9 per 100 person-years vs. 5.1 per 100 person-years) had a numerically lower post-matching thromboembolic event rate ( Table 2 ). Similarly, edoxaban versus apixaban had a numerically lower IS event rate (3.8 per 100 person-years vs. 5.0 per 100 person-years); however, the SE event rate was similar in the overall population ( Table 2 ). Event rates for any MB, major GI bleeding, ICH, and other MB were also similar for edoxaban versus apixaban in the overall population ( Table 2 ).

**Table 2:** Post-matching clinical outcomes at 12 months among patients overall and those aged ≥80 years: Edoxaban versus apixaban

Annualized rate for patients overall a		
**Event**		
	**Edoxaban (** ***n*** ** = 3,187)**	**Apixaban (** ***n*** ** = 3,187)**
**Effectiveness**		
IS or SE	3.9	5.1
IS	3.8	5.0
SE	0.1	0.1
**Safety**		
Any MB	0.9	0.8
Major GI bleeding	0.4	0.2
ICH	0.2	0.5
Other MB	0.2	0.1
** Annualized rate for patients ≥80 years of age a**		
	**Edoxaban (** ***n*** ** = 1,566)**	**Apixaban (** ***n*** ** = 1,566)**
**Effectiveness**		
IS or SE	3.9	6.3
IS	3.7	6.2
SE	0.2	0.1
**Safety**		
Any MB	1.3	1.2
Major GI bleeding	0.7	0.3
ICH	0.4	0.5
Other MB	0.2	0.4

Abbreviations: CCI, Charlson Comorbidity Index; CHA _2_ DS _2_ -VASc, Congestive heart failure, Hypertension, Age ≥75 (doubled), Diabetes, Stroke (doubled), Vascular disease, Age 65 to 74, and Sex category (female); GI, gastrointestinal; ICH, intracranial hemorrhage; IS, ischemic stroke; MB, major bleeding; SE, systemic embolism; SMD, standardized mean difference.

Propensity score matching was performed to adjust for confounding factors by separately matching the edoxaban cohort to the apixaban cohort 1:1 based on sex, age at index prescription, CHA _2_ DS _2_ -VASc score, CCI score, bleeding history, and concomitant medications. The balance between groups was assessed using SMDs.

a Data are shown as event rate per 100 person-years.

**Fig. 2:**
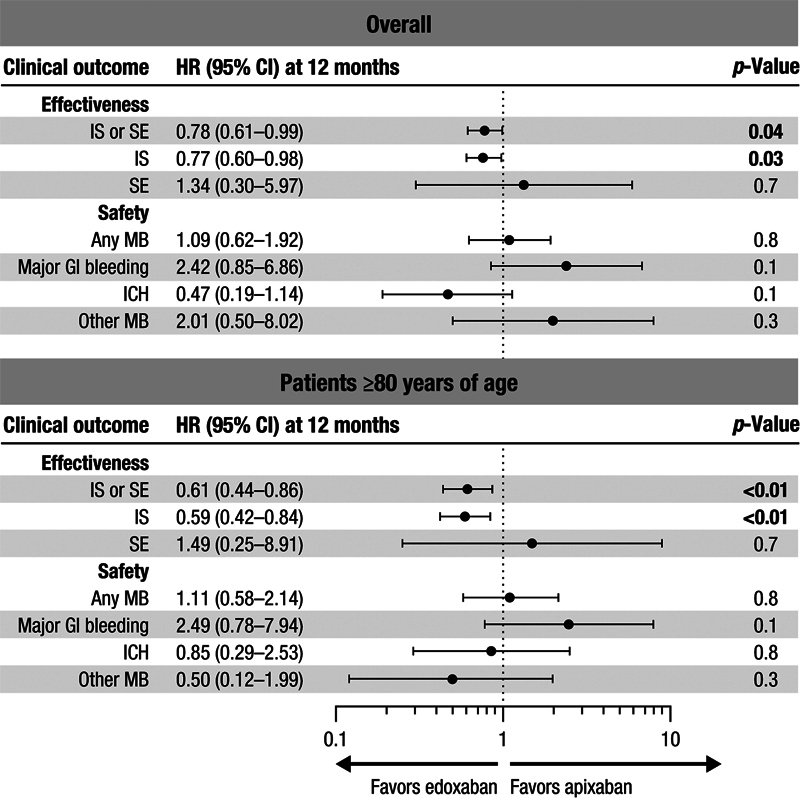
Post-matching clinical outcome hazard ratios at 12 months among patients with AF treated with edoxaban versus apixaban overall and for those aged ≥80 years. Propensity score matching was performed to adjust for confounding factors by separately matching the edoxaban cohort to the apixaban cohort 1:1 based on sex, age at index prescription, CHA _2_ DS _2_ -VASc score, CCI score, bleeding history, and concomitant medications. The balance between groups was assessed using SMDs. AF, atrial fibrillation; CCI, Charlson Comorbidity Index; CHA _2_ DS _2_ -VASc, Congestive heart failure, Hypertension, Age ≥75 (doubled), Diabetes, Stroke (doubled), Vascular disease, Age 65 to 74, and Sex category (female); CI, confidence interval; GI, gastrointestinal; HR, hazard ratio; ICH, intracranial hemorrhage; IS, ischemic stroke; MB, major bleeding; SE, systemic embolism; SMD, standardized mean difference.

The post-matching 12-month HR risks of clinical outcomes after adjusting for potential confounders identified statistically significant differences in thromboembolic event rates for edoxaban versus apixaban. The post-matching risk (HR, 95% CI) of IS or SE was significantly lower for edoxaban versus apixaban (0.78, 0.61–0.99; *p* = 0.04), driven by the lower risk of IS (0.77, 0.60–0.98; *p* = 0.03; Fig. 2 ). However, edoxaban compared with apixaban had no significant difference in the risk of any MB, major GI bleeding, ICH, or other MB ( Fig. 2 ).

#### Patients Aged ≥80 Years

For the population aged ≥80 years, edoxaban (3.9 per 100 person-years) compared with apixaban (6.3 per 100 person-years) had a numerically lower post-matching thromboembolic event rate ( Table 2 ). Similarly, the IS event rate was also numerically lower for edoxaban versus apixaban (3.7 per 100 person-years vs. 6.2 per 100 person-years); however, the SE event rate was similar for edoxaban versus apixaban ( Table 2 ). Post-matching, edoxaban versus apixaban had similar event rates for any MB, major GI bleeding, ICH, and other MB ( Table 2 ).

Consistent with observations in the overall population, the post-matching 12-month HR risks of clinical outcomes after adjusting for potential confounders identified statistically significant differences in thromboembolism for edoxaban versus apixaban in patients aged ≥80 years. The post-matching risk (HR, 95% CI) of IS or SE (0.61, 0.44–0.86; *p* < 0.01) and IS (0.59, 0.42–0.84; *p* < 0.01) was significantly lower for edoxaban compared with apixaban ( Fig. 2 ). However, the post-matching risks of any MB, major GI bleeding, ICH, and other MB were not significantly different for edoxaban versus apixaban ( Fig. 2 ).

#### Patients Aged <80 Years

For the population aged <80 years, edoxaban (3.9 per 100 person-years) had a numerically lower post-matching thromboembolic event rate compared with apixaban (4.2 per 100 person-years; Supplementary Table S4 ). Post-matching, edoxaban and apixaban had similar event rates for any MB.

In contrast with observations in the overall population and in the subgroup of patients aged ≥80 years, the post-matching 12-month HR risks of clinical outcomes after adjusting for potential confounders identified no statistically significant differences in thromboembolism for edoxaban versus apixaban in patients aged <80 years ( Supplementary Fig. S1 ). The post-matching HR (95% CI) of IS or SE was 0.94 (0.66–1.34) for edoxaban versus apixaban ( *p* = 0.72). There were no significant differences in the risk of any MB, major GI bleeding, ICH, or other MB between edoxaban and apixaban (all *p* > 0.05).

## Discussion

Older patients with AF, particularly those aged ≥80 years, represent a high-risk group for both thromboembolic and bleeding complications. 18 19 Managing anticoagulation in this population is challenging due to age-related physiological changes, comorbidities, and frequent polypharmacy. Therefore, selecting an oral anticoagulant that balances efficacy and safety while minimizing complexity is critical.

This retrospective database study compared the effectiveness and safety outcomes of newly prescribed edoxaban versus apixaban among patients with AF in Italy. A key highlight of this study is the analysis of outcomes in a large subgroup of patients aged ≥80 years (4,253/8,444, 50.4%), who have previously shown a heightened risk of any stroke 18 and bleeding. 19 20 In this group, consistent with the overall population, edoxaban was associated with a significantly lower risk of IS or SE compared with apixaban, with no difference in MB risk. Among patients aged <80 years, there were no significant differences in effectiveness or safety outcomes between edoxaban and apixaban, indicating that the differences observed in the overall population were largely driven by patients aged ≥80 years. This study was strengthened by a large sample size of patients with AF who had a new DOAC prescription.

Several factors support the use of edoxaban in older patients aged ≥80 years. First, its dose reduction criteria are simpler and more consistent compared with apixaban, 25 which helps minimize dosing errors and reduces the risk of inappropriate under- or overdosing. 26 Second, edoxaban's favorable drug–drug interaction profile is particularly beneficial for older adults, who often require multiple medications and are at higher risk of adverse interactions due to polypharmacy. 22

Although no RCTs have directly compared edoxaban and apixaban, several meta-analyses and real-world studies report similar safety profiles between edoxaban and apixaban, particularly regarding MB risk. 27 28 29 30 31 A small number of real-world analyses have investigated the safety of DOACs in elderly (≥75 or ≥80 years of age) populations, and only one directly compared edoxaban with apixaban. 30 31 32 33 In a network meta-analysis by Lee et al, 32 edoxaban and apixaban had a lower relative risk of MB compared with other DOACs in patients aged ≥80 years. A population-based retrospective cohort study using data from the United Kingdom Clinical Practice Research Datalink by Chiv et al 33 found that edoxaban was associated with a higher risk of MB compared with apixaban, mainly driven by major GI bleeding, in elderly patients (≥80 years) with AF between 2015 and 2021. However, Chiv et al characterized MB as “hospitalization for any bleeding,” a definition that, in contrast to the definition proposed by Cunningham et al, 24 is not limited to critical sites, and this may overestimate the incidence of MB events.

In contrast to the results of Chiv et al, a retrospective cohort study from Chan et al 30 comparing the safety of DOACs in patients with AF from the National Health Insurance Research Database (mean age: 75 years) found no significant differences in the risk of bleeding events between patients receiving edoxaban ( *n* = 4,577) versus apixaban ( *n* = 9,952). Similarly, a retrospective cohort study from Lee et al 31 comparing the safety of DOACs in patients with AF from the Health Insurance Review and Assessment database from January 2015 to December 2017 found no significant difference in the risk of MB between patients aged ≥75 years receiving edoxaban ( *n* = 15,496) versus apixaban ( *n* = 22,177; HR, 95% CI; 0.96, 0.75–1.2). Overall, while some studies suggest differences in bleeding risk between edoxaban and apixaban, most real-world evidence, including retrospective cohort studies and meta-analyses, indicates no significant difference in MB outcomes between the two, particularly in patients aged ≥80 years.

In the current study, the incidence of thromboembolic events for patients on edoxaban was similar in the subgroup ≥80 years of age, the subgroup <80 years of age, and the overall population, while the occurrence of thromboembolic events for patients on apixaban was more common in the subgroup ≥80 years of age than in the subgroup <80 years of age or the overall population. Further, compared with apixaban, edoxaban had a significantly lower rate of IS or SE in patients ≥80 years of age. Both the current study and a previous meta-analysis 32 reported a numerically lower risk of thromboembolism with edoxaban compared with apixaban; however, in contrast to that meta-analysis, the current study demonstrated a statistically significant difference.

Collectively, these findings indicate that edoxaban achieves an appropriate balance between efficacy and safety in older patients with AF, facilitating effective stroke prevention while maintaining a comparable bleeding risk profile to apixaban. The combination of streamlined dose adjustment criteria and a lower propensity for clinically relevant drug–drug interactions further supports the suitability of edoxaban for anticoagulation management in this vulnerable population.

## Study Limitations

In this study, propensity score matching was used to reduce the potential bias from comparing edoxaban with that of apixaban use. Nonetheless, unobserved residual confounding effects may remain after matching patients based on available baseline patient demographics and characteristics. Potential confounders may include clinic prescribing patterns and unavailable laboratory results. Body weight and renal function data were not available in the database; thus, adequate dosing could not be determined, and these factors could not be adjusted for propensity score matching. Data to calculate days of supply, which are needed to measure adherence for each group, were not available in the database; therefore, differences in outcomes may in part be attributable to differences in adherence, which may vary between once-daily and twice-daily regimens. 34 35 36 Additional bias may be introduced by missing values, coding errors, and the inability to clinically verify database records. The results of this study cannot be generalized to patients who previously used a DOAC or VKA. As this study was conducted to analyze data from patients with AF from an Italian database, the results cannot be generalized to patients from other countries. The distribution of patient characteristics and clinical outcomes may have been impacted by the lack of distinction between individual doses of edoxaban or apixaban (i.e., edoxaban 30 vs. 60 mg or apixaban 2.5 vs. 5 mg) in our study. Finally, actual patient adherence to drug use as prescribed could not be verified, given that the database is formed on the interactions of health care practitioners with patients.

## Conclusion

In routine clinical practice in Italy, patients with AF, including those ≥80 years of age, prescribed edoxaban versus apixaban had a significantly lower risk of IS or SE with a similar risk of MB. These findings may help optimize treatment recommendations and support physicians in making informed DOAC prescribing decisions.

## Notes

**What is Known About This Topic?**

- In Italy, DOACs are increasingly prescribed over VKAs to prevent thromboembolism in patients with AF.
- No head-to-head RCT comparing edoxaban and apixaban is available, nor is one expected, leaving real-world evidence as the primary source of data to inform clinical decision-making.
- Older patients (≥80 years) with AF face higher risks of any stroke and bleeding, with age-related pharmacokinetic and pharmacodynamic changes potentially influencing anticoagulant effects.

**What Does This Paper Add?**

- Among Italian patients with AF overall and those ≥80 years of age who received a new edoxaban or apixaban prescription between 2016 and 2021, the risk of IS or SE was significantly lower for edoxaban versus apixaban.
- Among patients with AF overall and those ≥80 years of age, the risk of MB was similar between edoxaban and apixaban.
- These findings may help optimize treatment recommendations and support physicians in making informed DOAC-prescribing decisions in Italy.
