# Supplementary material for: Edoxaban versus Apixaban Outcomes Differences in 8,444 Patients with Atrial Fibrillation from Italy: A Real-World Use Comparison
Source: TH Open. 2026 Mar 23;10:a28229746. doi: 10.1055/a-2822-9746 (PMC13305731; doi:10.1055/a-2822-9746)
Supplement: Supplementary file 1 — Supplementary Material [file 10-1055-a-2822-9746-s26020010.pdf]

Thieme

Supplementary Material

Supplementary Table 1. Baseline demographics and clinical characteristics among patients aged <80 years before matching.[a,b]

	Edoxaban (n = 1622)	Apixaban (n = 2569)	*p*-Value[b]
**Demographics**			
**Age,** years			
Mean (SD)	70.6 (7.4)	70.7 (7.6)	0.7
Age category			0.1
≥64	275 (17.0)	445 (17.3)	
65-74	769 (47.4)	1137 (44.3)	
≥75	578 (35.6)	987 (38.4)	
**Sex**			
Female	769 (47.4)	1163 (45.3)	0.2
**Clinical characteristics**			
**$CHADS_2$ score,** mean (SD)	1.6 (0.9)	1.7 (0.9)	**0.003**
**CHA_2DS_2-VASc score,** mean (SD)	3.0 (1.2)	3.0 (1.2)	**0.03**
**CCI score**			
Mean (SD)	0.7 (1.1)	0.8 (1.1)	**0.03**
CCI score category			**0.0005**
0	958 (59.1)	1390 (54.1)	
1	409 (25.2)	701 (27.3)	
2	134 (8.3)	301 (11.7)	
>2	121 (7.5)	177 (6.9)	
**Hypertension**	1464 (90.3)	2353 (91.6)	0.1
**Diabetes mellitus**	309 (19.1)	554 (21.6)	0.05
**Vascular disease**	112 (6.9)	248 (9.7)	**0.002**
Coronary artery disease	79 (4.9)	185 (7.2)	**0.003**
Peripheral artery disease	44 (2.7)	79 (3.1)	0.5
**Stroke/transient ischemic attack**	62 (3.8)	118 (4.6)	0.2
**Congestive heart failure**	99 (6.1)	157 (6.1)	1.0
**Cerebral vascular disease**	120 (7.4)	236 (9.2)	**0.04**
**Chronic pulmonary disease**	134 (8.3)	240 (9.3)	0.2
**Cancer**	97 (6.0)	131 (5.1)	0.2
**Bleeding history or predisposition**	8 (0.5)	22 (0.9)	0.2
**Medications**			
Antiplatelet	136 (8.4)	301 (11.7)	**0.0006**
NSAIDs	148 (9.1)	225 (8.8)	0.7
Proton pump inhibitors	703 (43.3)	1156 (45.0)	0.3
ACEI-ARB	679 (41.9)	1200 (46.7)	**0.002**
Amiodarone	168 (10.4)	273 (10.6)	0.8
Beta-blocker	968 (59.7)	1569 (61.1)	0.4
Statins	603 (37.2)	977 (38.0)	0.6

[a]Data are shown as n (%) unless otherwise noted.

[b]Baseline clinical characteristics <5% are not shown.

TH Open | DOI 10.1055/a-2822-9746 | © The Author(s).

[c] *P*-values indicate comparison to the edoxaban cohort. Bolded values indicate $P<0.05$.

ACEI-ARB, angiotensin-converting enzyme inhibitor-angiotensin receptor blocker; AF, atrial fibrillation; CCI, Charlson Comorbidity Index; $CHADS_2$, Congestive heart failure, Hypertension, Age ≥75, Diabetes, Stroke (doubled); CHA_2DS_2-VASc, Congestive heart failure, Hypertension, Age ≥75 (doubled), Diabetes, Stroke (doubled), Vascular disease, Age 65 to 74, and Sex category (female); NSAID, nonsteroidal anti-inflammatory drug; SD, standard deviation.

TH Open | DOI 10.1055/a-2822-9746 | © The Author(s).

Supplementary Table 2. Baseline demographics and clinical characteristics after matching among patients overall, those aged ≥80 years, and those aged <80 years.[a]

	Overall			Aged ≥80 years			Aged <80 years		
	Edoxaban (n = 3187)	Apixaban (n = 3187)	SMD[b]	Edoxaban (n = 1566)	Apixaban (n = 1566)	SMD[b]	Edoxaban (n = 1621)	Apixaban (n = 1621)	SMD[b]
**Age,** years									
Mean (SD)	78.1 (9.8)	78.2 (9.9)	0.01	85.9 (4.3)	85.7 (4.3)	0.05	70.6 (7.4)	70.8 (7.4)	0.03
**Sex**			0.01			0.01			0.04
Female	1709 (53.6)	1690 (53.0)		625 (39.9)	636 (40.6%)		852 (52.6)	848 (52.3)	
**CHA_2DS_2-VASc score**									
Mean (SD)	3.5 (1.2)	3.5 (1.2)	<0.0001	4.0 (0.9)	4.0 (0.9)	0.02	3.0 (1.2)	3.0 (1.2)	0.01
CHA_2DS_2-VASc category									
0	20 (0.6)	20 (0.6)		0	0		20 (1.2)	20 (1.2)	
1	157 (4.9)	158 (5.0)		87 (5.6)	112 (7.2)		157 (9.7)	146 (9.0)	
2	434 (13.6)	418 (13.1)		1003 (64.0)	997 (63.7)		394 (24.3)	387 (23.9)	
3	924 (29.0)	944 (29.6)		368 (23.5)	336 (21.5)		541 (33.4)	541 (33.4)	
4	1135 (35.6)	1144 (35.9)		81 (5.2)	99 (6.3)		361 (22.3)	405 (25.0)	
5	384 (12.1)	366 (11.5)		27 (1.7)	21 (1.3)		110 (6.8)	93 (5.7)	
6	104 (3.3)	111 (3.5)		0	1 (0.1)		33 (2.0)	25 (1.5)	
7	27 (0.9)	22 (0.7)		0	0		4 (0.3)	3 (0.2)	
8	2 (0.1)	4 (0.1)		0	0		1 (0.1)	1 (0.1)	
9	0	0		0	0		0	0	
**CCI score**									
Mean (SD)	0.7 (1.1)	0.7 (1.1)	0.01	0.8 (1.1)	0.8 (1.1)	<0.0001	0.7 (1.1)	0.7 (1.1)	0.01
CCI score category									
0	1774 (55.7)	1798 (56.4)		817 (52.2)	823 (52.6)		958 (59.1)	962 (59.3)	
1	848 (26.6)	835 (26.2)		439 (28.0)	446 (28.5)		409 (25.2)	404 (24.9)	
2	325 (10.2)	322 (10.1)		191 (12.2)	169 (10.8)		134 (8.3)	161 (9.9)	
3	153 (4.8)	141 (4.4)		78 (5.0)	79 (5.0)		75 (4.6)	49 (3.0)	
4	50 (1.6)	55 (1.7)		20 (1.3)	28 (1.8)		29 (1.8)	29 (1.8)	
5	19 (0.6)	19 (0.6)		13 (0.8)	13 (0.8)		6 (0.4)	7 (0.4)	
6	13 (0.4)	9 (0.3)		7 (0.5)	5 (0.3)		6 (0.4)	3 (0.2)	
7	3 (0.1)	6 (0.2)		0	3 (0.2)		3 (0.2)	3 (0.2)	
8	2 (0.1)	2 (0.1)		1 (0.1)	0		1 (0.1)	3 (0.2)	
10	0	0		0	0		0	0	
**Bleeding history or predisposition**	19 (0.6)	13 (0.4)	0.03	11 (0.7)	12 (0.8)	0.01	8 (0.5)	11 (0.7)	0.02
**Medications**									
Antiplatelet	323 (10.1)	316 (9.9)	0.01	187 (11.9)	168 (10.7)	0.04	135 (8.3)	122 (7.5)	0.03
NSAIDs	275 (8.6)	232 (7.3)	0.05	127 (8.1)	103 (6.6)	0.06	148 (9.1)	134 (8.3)	0.03
Proton pump inhibitors	1446 (45.4)	1453 (45.6)	0.004	743 (47.5)	752 (48.0)	0.01	702 (43.3)	693 (42.8)	0.01
ACEI-ARB	1361 (42.7)	1374 (43.1)	0.01	682 (43.6)	669 (42.7)	0.02	678 (41.8)	701 (43.2)	0.03
Amiodarone	294 (9.2)	261 (8.2)		126 (8.1)	126 (8.1)		168 (10.4)	155 (9.6)	
Beta-blocker	1828 (57.4)	1873 (58.8)	0.03	860 (54.9)	864 (55.2)	0.01	967 (59.7)	962 (59.3)	0.01
Statins	1124 (35.3)	1104 (34.6)	0.01	522 (33.3)	522 (33.3)	<0.0001	602 (37.1)	595 (36.7)	0.01

TH Open | DOI 10.1055/a-2822-9746 | © The Author(s).

Propensity score matching was performed to adjust for confounding factors by separately matching the edoxaban cohort to the apixaban cohort 1:1 based on sex, age at index prescription, CHA_2DS_2-VASc score, CCI score, bleeding history, and concomitant medications. The balance between groups was assessed using SMDs.

[a]Data are shown as n (%) unless otherwise noted.

[b]SMDs indicate comparison between the edoxaban and apixaban cohorts. Adequate balance was achieved for all covariates included in the propensity score matching (SMD ≤0.1).

ACEI-ARB, angiotensin-converting enzyme inhibitor-angiotensin receptor blocker; CCI, Charlson Comorbidity Index; CHA_2DS_2-VASc, Congestive heart failure, Hypertension, Age ≥75 (doubled), Diabetes, Stroke (doubled), Vascular disease, Age 65 to 74, and Sex category (female); NSAID, nonsteroidal anti-inflammatory drug; SD, standard deviation; SMD, standardized mean difference.

TH Open | DOI 10.1055/a-2822-9746 | © The Author(s).

Supplementary Table 3. Pre-matching clinical outcomes at 12 months among patients overall and those aged ≥80 years: edoxaban vs apixaban.

Event	Annualized rate for patients overall[a]	
	**Edoxaban (n = 3188)**	**Apixaban (n = 5256)**
**Effectiveness**		
IS or SE	3.9	5.5
IS	3.8	5.4
SE	0.1	0.1
**Safety**		
Any MB	0.9	1.0
Major GI bleeding	0.4	0.3
ICH	0.2	0.5
Other MB	0.2	0.3
	**Annualized rate for patients ≥80 years of age[a]**	
	**Edoxaban (n = 1566)**	**Apixaban (n = 2687)**
**Effectiveness**		
IS or SE	3.9	6.8
IS	3.7	6.6
SE	0.2	0.1
**Safety**		
Any MB	1.3	1.3
Major GI bleeding	0.7	0.3
ICH	0.4	0.7
Other MB	0.2	0.4

[a]Data shown as event rate per 100 person-years.

GI, gastrointestinal; ICH, intracranial hemorrhage; IS, ischemic stroke; MB, major bleeding; SE, systemic embolism.

TH Open | DOI 10.1055/a-2822-9746 | © The Author(s).

Supplementary Table 4. Pre- and post-matching clinical outcomes among patients aged <80 years at 12 months: edoxaban vs apixaban

Event	Annualized rate for patients <80 years of age[a]			
	Pre-matching		Post-matching	
	Edoxaban (n = 1622)	Apixaban (n = 2569)	Edoxaban (n = 1621)	Apixaban (n = 1621)
**Effectiveness**				
IS or SE	4.0	4.2	3.9	4.2
IS	3.9	4.1	3.9	4.0
SE	0.1	0.1	0.1	0.1
**Safety**				
Any MB	0.4	0.6	0.4	0.5
Major GI bleeding	0.1	0.3	0.1	0.4
ICH	0.1	0.3	0.1	0.1
Other MB	0.2	0.1	0.2	0.1

Propensity score matching was performed to adjust for confounding factors by separately matching the edoxaban cohort to the apixaban cohort 1:1 based on sex, age at index prescription, CHA_2DS_2-VASc score, CCI score, bleeding history, and concomitant medications. The balance between groups was assessed using SMDs.

CCI, Charlson Comorbidity Index; CHA_2DS_2-VASc, Congestive heart failure, Hypertension, Age ≥75 (doubled), Diabetes, Stroke (doubled), Vascular disease, Age 65 to 74, and Sex category (female); GI, gastrointestinal; ICH, intracranial hemorrhage; IS, ischemic stroke; MB, major bleeding; SE, systemic embolism; SMD, standardized mean difference.

TH Open | DOI 10.1055/a-2822-9746 | © The Author(s).

Supplementary Figure 1. Post-matching clinical outcome hazard ratios at 12 months among patients with AF treated with edoxaban vs apixaban aged <80 years.

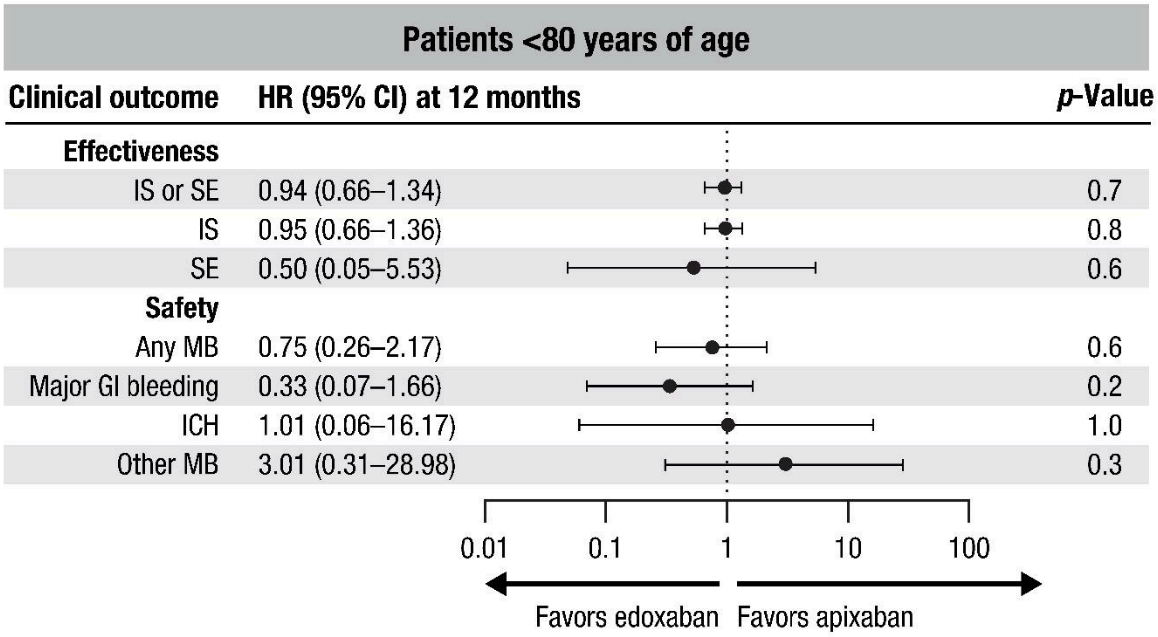

Propensity score matching was performed to adjust for confounding factors by separately matching the edoxaban cohort to the apixaban cohort 1:1 based on sex, age at index prescription, CHA_2DS_2-VASc score, CCI score, bleeding history, and concomitant medications. The balance between groups was assessed using SMDs. AF, atrial fibrillation; CCI, Charlson Comorbidity Index; CHA_2DS_2-VASc, Congestive heart failure, Hypertension, Age ≥75 (doubled), Diabetes, Stroke (doubled), Vascular disease, Age 65 to 74, and Sex category (female); CI, confidence interval; GI, gastrointestinal; HR, hazard ratio; ICH, intracranial hemorrhage; IS, ischemic stroke; MB, major bleeding; SE, systemic embolism; SMD, standardized mean difference.

TH Open | DOI 10.1055/a-2822-9746 | © The Author(s).
